# Regulation of Adult Neurogenesis in Mammalian Brain

**DOI:** 10.3390/ijms21144869

**Published:** 2020-07-09

**Authors:** Maria Victoria Niklison-Chirou, Massimiliano Agostini, Ivano Amelio, Gerry Melino

**Affiliations:** 1 Centre for Therapeutic Innovation (CTI-Bath), Department of Pharmacy & Pharmacology, University of Bath, Bath BA2 7AY, UK; mvnc20@bath.ac.uk; 2 Blizard Institute of Cell and Molecular Science, Barts and the London School of Medicine and Dentistry, Queen Mary University of London, London E1 2AT, UK; 3 Department of Experimental Medicine, TOR, University of Rome “Tor Vergata”, 00133 Rome, Italy; m.agostini@med.uniroma2.it (M.A.); ivano.amelio@uniroma2.it (I.A.); 4 School of Life Sciences, University of Nottingham, Nottingham NG7 2HU, UK

**Keywords:** neurogenesis, transcription factors, epigenetic modification, metabolism, p73, p53

## Abstract

Adult neurogenesis is a multistage process by which neurons are generated and integrated into existing neuronal circuits. In the adult brain, neurogenesis is mainly localized in two specialized niches, the subgranular zone (SGZ) of the dentate gyrus and the subventricular zone (SVZ) adjacent to the lateral ventricles. Neurogenesis plays a fundamental role in postnatal brain, where it is required for neuronal plasticity. Moreover, perturbation of adult neurogenesis contributes to several human diseases, including cognitive impairment and neurodegenerative diseases. The interplay between extrinsic and intrinsic factors is fundamental in regulating neurogenesis. Over the past decades, several studies on intrinsic pathways, including transcription factors, have highlighted their fundamental role in regulating every stage of neurogenesis. However, it is likely that transcriptional regulation is part of a more sophisticated regulatory network, which includes epigenetic modifications, non-coding RNAs and metabolic pathways. Here, we review recent findings that advance our knowledge in epigenetic, transcriptional and metabolic regulation of adult neurogenesis in the SGZ of the hippocampus, with a special attention to the p53-family of transcription factors.

## 1. Introduction

The development of the mammalian central nervous system (CNS) is a spatial and temporal regulated process that evolves from a small number of cells that proliferate, acquire regional identities and give rise to different cell types [1]. These cells have been classified as neural stem cells (NSCs) and have the capability to produce identical NSCs progeny through symmetric cell division (self-renewal) and to differentiate into specialized brain cell types such as neurons, astrocytes and oligodendrocytes [2,3,4]. Under physiological conditions, adult neurogenesis is restricted in neurogenic niches that are localized in two different regions of the brain, the subventricular zone (SVZ) of the lateral ventricle where new neurons are generated and then migrate to the olfactory bulb (OB), and the subgranular zone (SGZ) of the dentate gyrus (DG) of the hippocampus [4,5,6].

Although the process of neurogenesis is complex and highly regulated, it can be divided into six different stages [7]. Stage 1 occurs 1–3 days after birth and is called the proliferation phase; during this, neuronal progenitor cells (NPCs) are capable of proliferation and multi-potential differentiation but are unable to self-renew. Stages 2–4 occur approximately 1 week after birth and are collectively called the differentiation phase; during this time, neuronal progenitors exit from the cell cycle and are committed to the neuronal lineage. After the commitment, immature neurons enter stage 5, known as the migration phase, to reach their final destination. This event occurs between 2 to 3 weeks after birth. Post-mitotic neurons start to extend their axonal projections and dendritic growth starts. Finally, stage 6 of adult neurogenesis occurs at approximately 4 weeks after birth and is the synaptic integration where newly generated neurons establish their synaptic contacts into the pre-existing circuits [8,9]. Overall, it takes about 2–4 months for indistinguishable adult-born neurons to fully integrate with surrounding cells and incorporate into the hippocampal circuits [10,11].

Multiple lines of evidence indicate that both extrinsic cues and intrinsic pathways are required for the regulation of this developmental process [8,12,13]. Among the intrinsic pathways, multilayered regulatory networks display connections between multiple complex transcription factors, epigenetic control, non-coding RNAs, signalling and metabolic pathways [14,15].

Understanding how neuronal differentiation is regulated in adulthood is crucial for understanding the pathogenesis of several disorders that affect the CNS including neurodegenerative diseases, psychiatric and neurodevelopmental disorders. The present review discusses and summarises the recent research on adult neurogenesis to improve our understanding of the transcriptional, epigenetic and metabolic regulation of neurogenesis in the human brain.

## 2. Epigenetic Regulation of Adult Neurogenesis in SGZ of the DG

**Figure 1:**
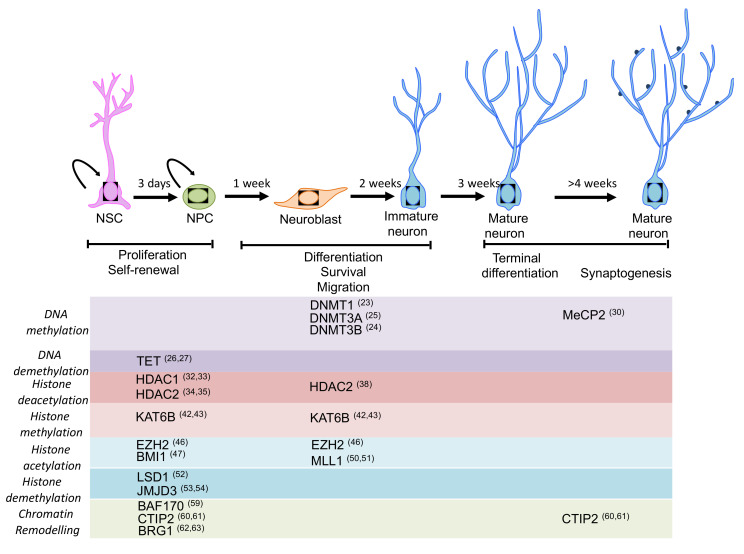
Schematic representation of the main stages of neurogenesis and the epigenetic machinery involved in the regulation of neurogenesis. Expression pattern of the main epigenetic regulators for which a function in adult neurogenesis has been described or proposed. Most of the regulators play an important role in self-renewal, proliferation and fate specification during neurogenesis (please see main text for details).

Epigenetic mechanisms such as DNA methylation, histone post-translational modifications and chromatin remodelling, are essential players in determining adult neuronal differentiation by regulation of gene expression [16]. Moreover, alterations (mutations of regulators and/or deregulations) of epigenetic mechanisms have critical implications in several human conditions, including neurological disorders, such as autism spectrum disorder, mental retardation and epilepsy. This highlights the important roles that epigenetic mechanisms exert for brain development and function [17,18]. See Figure 1 for a schematic view.

### 2.1. DNA Methylation

Methylation of the cytosine (5mC) of the dinucleotide CpG islands within the promoters of genes has been the first described epigenetic modification [19,20]. CpG islands are short stretches of palindromic DNA with the sequence “CpG” that code for cytosine (C) and guanine (G) nucleotides with the “p” representing the linking phosphate. Typically, methylation of the CpG islands is associated with their transcription repression [21]. Moreover, 5mC is enzymatically mediated by DNA methyl transferases (DNMTs), while removal of the methyl group is triggered by the ten-eleven translocation (TET) family [21].

DNA methylation is a key process by which pluripotency genes are silenced during the neural induction of embryonic stem cells to NSCs, supporting the importance of DNA methylation during neurogenesis [22]. While DNMT1 controls the timing of astrogliogenesis through regulation of JAK-STAT signalling [23], DNMT3A and DNMT3B are crucial for neuron specification. DNMT3A is expressed in neurons and its expression increases postnatally. Ablation of DNMT3A in the brain (using Nestin-Cre system) results in an impaired postnatal neurogenesis, reduced number of neurons and mice death in early postnatal stages [24]. During neurogenesis, DNMT3B is expressed in NPCs and its deletion results in an accelerated neuronal maturation as shown by an upregulation of several neuronal genes including NeuroD1, Map2m and Ncam1 [25].

TET family enzymes are responsible for activating the erasure of the methyl group from 5mC. Deletion of Tet genes in mice using the Nestin-Cre ERT2 system leads to a reduction of the number of NPCs in the adult SGZ, and impairment in learning and memory [26]. At a molecular level, this phenotype is associated with the deregulation of several genes involved in NPCs proliferation such as, galanin (Gal), chondroitin sulphate proteoglycan 4 (Cspg4) and neuroglobin (Ngb). Moreover, neuronal activity in the adult mouse hippocampus can stimulate neurogenesis [27]. In this context, growth arrest and DNA-damage-inducible protein 45β (GADD45β) promote adult hippocampal neurogenesis by reducing the degree of DNA methylation of the promoter and therefore increasing the expression of key neuronal genes, including Bdnf and Fgf1. This results in promoting NPCs proliferation and the development of new neurons [28,29]. Moreover, deficiency of MeCp2 hinders maturation and impairs dendritic and spine morphogenesis of new neurons [30]. Hence, DNA methylation has a critical role in NPCs maintenance and fate specification in adult neurogenesis.

### 2.2. Histone Post-Translational Modifications

The chemical covalent modifications of the histone, including acetylation, methylation, ubiquitination, phosphorylation, ribosylation, and SUMOylation, play a key role in regulating the chromatin state [31]. In general, acetylation of specific amino acid residues and di- or tri-methylation of histone H3 lysine 4 (H3K4) are associated with active transcription. On contrary, di- or tri-methylation of H3K9 and H3K27 are stable repressive marks. Histone acetyltransferases (HATs) and histone deacetylases (HDACs) are the enzymes that regulate acetylation (transcriptional activation) or deacetylation (transcriptional silencing) of the histone, respectively.

HDACs are the best-studied histone modifiers in the context of neuronal differentiation [32]. A comprehensive gene expression analysis of the 11 isoforms of HDACs in rat brain show a ubiquitous expression of class I HDACs in the CNS, while class II HDACs show a tissue-specific pattern of expression, suggesting that HDAC isoforms expression may also be developmentally regulated [33]. Moreover, HDAC1 is mainly expressed in NSCs, while HDAC2 in mature neurons [34]. HDAC1 and HDAC2 have essential and redundant roles during brain development [35]. Indeed, deletion of HDAC1 or HDAC2 in neuronal precursors (using human glial fibrillary acidic protein (hGFAP)-Cre system) shows no obvious phenotypes, while combined deletion in developing neurons results in severe abnormalities in brain formation including, loss of hippocampal structure and disorganisation of cortical neurons. These abnormalities are mainly linked to a failure of neuronal precursors to differentiate into mature neurons and to excessive cell death [36,37]. HDAC2 has an important role in adult brain. Indeed, Nestin-Cre mediated deletion of HDAC2 shows that neurons derived from adult neurogenesis die at a specific maturation stage. However, brain development and adult stem cell fate are normal [38]. Nevertheless, embryonic neurogenesis is not affected by the loss of HDAC2 catalytic activity, possibly because HDAC1 can compensate for the lack of HDAC2 [35]. The class II HDAC3 and HDAC5 are highly expressed in neuronal stem/progenitor cells and have also been implicated in neuronal differentiation by regulating their proliferation and differentiation. Conditional deletion of HDAC3 in neurons (calcium/calmodulin-dependent protein kinase II (CaMKII)-Cre system) and glia (Nestin-Cre system) result in a disorganisation of the neocortex and cerebellum. These mice show also perinatal death [39]. Apart from deacetylation of histone proteins, HDAC family members (HDAC 1, 3, 6 and 9) by deacetylate components of the microtubule and actin cytoskeleton, regulate neurite formation, dendrite and axon growth [40,41].

Opposite to the histone deacetylation, the reverse reaction (acetylation) that is catalysed by HAT is also involved in neurogenesis. KAT6B, which is highly expressed in the adult SVZ, plays an important role in adult neurogenesis [42]. KAT6B deficient mice (Querkopf^gt/gt^ mutant mice) show reduction in the number of NSCs and in the migrating neuroblasts in the rostral migratory stream [43].

Histone methylation/demethylation is associated with either activation or repression of transcription, depending on the position of methylated residues and on the number of methyl groups added [44]. The reaction of histone methylation is catalysed by lysine methyltransferases (KMTs). The well-known PcG repressive complex (PRC) and Trithorax active complex (TRXG) are involved in the regulation of adult NPCs and differentiated neurons [45]. Specifically, the methyltransferase component of PcG Enhancer of zeste homologue 2 (*Ezh2*) is expressed both in the SVZ and SGZ of adult mice. Conditional deletion (using hGFAP-Cre system) of Ezh2 in cerebellar granule cell layer, hippocampal dentate gyrus and SVZ NSCs, results in a reduced neurogenesis in both the DG and olfactory bulb [46]. This, at least in part, is linked to reduced cell proliferation. Mechanistically, Ezh2 is critical for the repression of genes involved in self-renewal and neuronal differentiation such as, Ink4a/Arf. BMI1 is another component of the PRC1 complex that is required for the self-renewal of the NSC but not for their survival and differentiation [47]. At a molecular level, constitutive deletion of BMI1 in mice results in the deregulation of p21-Rb pathway in NSC [48,49].

Mixed-lineage leukaemia 1 (MLL1) is a histone methyltransferase, member of the TRXG complex that regulates neurogenesis in the mouse postnatal brain [50]. In particular, MLL1 depletion in NSCs, using hGFAP-Cre system results in a severe impairment of neuronal differentiation, which is due to the loss of distal-less homeobox 2 (Dlx2) expression. Interestingly, deletion of MLL1 is associated with an increase of H3K27me3 that impairs the proper activation of DLX2 gene. More recently, MLL1 mainly regulates neuronal genes, including brain-specific POU-box gene, Brn4 [51].

Lysine-specific demethylase 1 (LSD1) is expressed in NSCs and is an important regulator of proliferation. Indeed, pharmacological and genetic inhibition (siRNA-expressing lentivirus) of LSD1 leads to a dramatic reduction in NSC proliferation [52]. Mechanistically, the orphan nuclear receptor TLX, also known as NR2E1TLX, recruits both LSD1 and HDAC5 on its target genes and this complex mediates gene repression.

Jumonji domain-containing protein 3 (JMJD3) is an H3K27me3-specific demethylase that is required for neuronal commitment by directly regulating the expression of Pax6, Nestin and Sox1 [53]. Moreover, JMJD3 is expressed in the adult SVZ and its conditional deletion (using hGFAP-Cre system) leads to an impaired OB neurogenesis by controlling the expression of several neurogenic genes (*Myt1, Slc32a1* and *Gjb6).* This is via interaction with the promoter regions as well as in conjunction with neurogenic enhancer elements (I12b), which in turn regulates Dlx2 [54].

Therefore, histone post-translational modifications play a critical role in neurodevelopment and embryonic and adult neurogenesis.

### 2.3. Chromatin Remodelling

Together with chemical modifications of histones and methylation of DNA, specific chromatin conformation is also required for the regulation of gene expression [55]. The presence of histones in the DNA poses a barrier to gene transcription, therefore the proper density and spacing of nucleosomes should be maintained. Specialized ATP-dependent chromatin-remodelling complexes including imitation switch (ISWI), chromodomain helicase DNA-binding (CHD), switch/sucrose non-fermentable (SWI/SNF also known as BRG1/BRM associated factor, BAF) and INO80 are responsible for nucleosome occupancy and composition. Chromatin remodelling complexes are formed by the assembly of different combinations of proteins and they have emerged as important regulators of neuronal development [56,57].

BAF is a multimeric complex comprised at least 10 to 15 subunits that bind to the promoter region of active genes involved in the maintenance of neuronal development by mainly influencing the expression of essential genes for neurogenesis, cell migration and functional integration of neurons [58]. In particular, components of BAF complex promote adult neurogenesis. Indeed, it has been shown that conditional deletion of the BAF170 subunit in adult brain by using hGFAP-Cre system results in the impairment of neuronal differentiation with premature generation of astrocytes [59].

Moreover, the Ctip2 subunit is mainly expressed in postmitotic granule neurons and is required for the proper development of the hippocampus. Conditional deletion (using Emx1-Cre system) of Ctip2 in adult hippocampus results in the reduction of the NPC pool and impairment of neuronal differentiation [60]. Moreover, Ctip2 subunit plays an important role in the regulation of neuronal survival and integration of new neurons into the pre-existing hippocampal circuits [61].

To further support the role of BAF complex during neurogenesis, the BRG1 subunit has been conditionally deleted (using Nestin-Cre system) in NSCs resulting in a premature neuronal differentiation [62]. Mechanistically, BRG1 interacts with Pax6, and the complex directly activates the neurogenic transcription factors such as Sox11, Nfib and Pou3f4 to Pax6 [63].

All together, these data indicate that epigenetic mechanisms are essential for maintaining neurogenesis throughout adult life.

## 3. Transcriptional Regulation of Adult Neurogenesis

**Figure 2:**
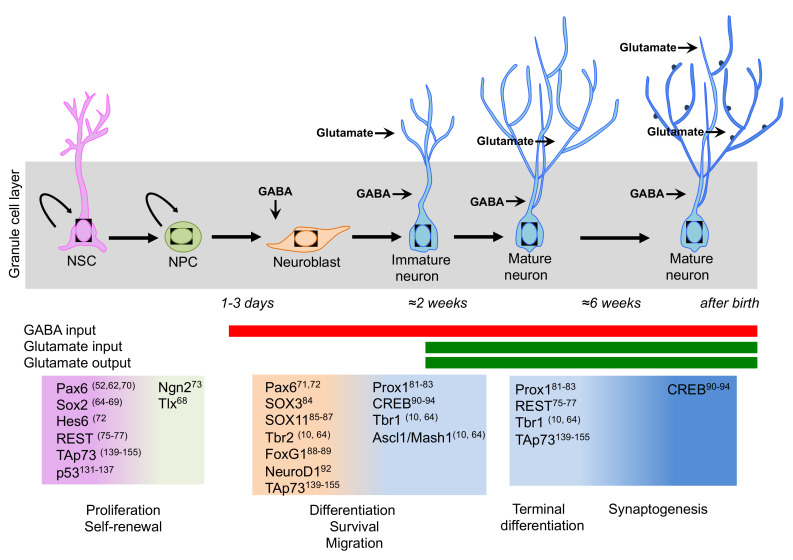
Schematic representation of the transcription factor network involved in neurogenesis in the adult hippocampus. A sequential and coordinated expression pattern of the neurogenic transcription factors is fundamental for the proper progression from neural stem cells (NSCs) to mature neurons (Please see main text for details). During neurogenesis, a switch takes place from GABA excitatory to GABA inhibitory and glutamate excitatory inputs.

Among the intrinsic signals that regulate the sophisticated intracellular networks for NSC/NPC maintenance and neuronal differentiation, the transcription factors have been the most studied and investigated components. Progression through the different developmental stages during adult neurogenesis is accompanied by the up- and down-regulation of several transcription factors. Thus, transcription factors are the pivotal regulators of gene expression and potential mediators of extrinsic signals [64]. Interestingly, this network is almost conserved during neurogenesis in the developing and adult brain. Here, we will discuss some key neurogenic transcription factors, as visualized in Figure 2, and we will summarise recent work on the well-established role of a p53 family in regulating neurogenesis.

### 3.1. Transcriptional Control of Maintenance and Cell Fate Decision of NSCs in Adult Hippocampus

Among the transcription factors involved in the regulation of adult hippocampal neurogenesis, the Sox family is the most extensively investigated [65]. Sox2 has an active role in neural stem cells renewal. However, recent findings have shown that some neurons and glia cells keep high Sox2 expression, which is necessary for their function including cellular morphology and connectivity [66]. Conditional deletion (using Nestin-Cre system) of Sox2 during embryonic development results in a complete absence of the DG by post-natal day 7 [67]. To further support this, when Sox2 is deleted by retrovirus injection into an adult hippocampus, a decline of the positive type-1 cells population is observed suggesting that Sox2 is required for NPC maintenance in the dentate gyrus. Mechanistically, Sox2 directly regulates the expression of Sonic hedgehog (Shh) pathway. In parallel, Sox2 must repress the expression of NeuroD1 in order to maintain the self-renewal capacity of NSCs and therefore preventing the progression of neurogenesis [68]. Moreover, Sox2 controls the expression of the nuclear orphan receptor Tlx, which in turns supports proliferation and self-renewal of adult NSCs in the DG [69]. On the other hand, Sox2 expression is regulated by the Notch/RBPJk signalling, which is required for self-renewal and expansion of NSCs in the adult hippocampus [70].

The paired box protein, Pax6 is a transcription factor that was discovered to be essential for eye development; Pax6 is also crucial for the maintenance of NSCs pool [71]. It is mainly present in NSCs and its expression also persists to some extent in progenitor cells [72]. Pax6 directly regulates the expression of a cohort of genes important for self-renewal and neuronal differentiation including *Hmga2, Cdk4, Gadd45g, Neurod1, Sstr2* and *Hes6*. Recently, the gene regulatory circuitry of Pax6 during neurogenesis has been mapped [73]. Among them, the pro-neural protein neurogenin 2 (Ngn2), regulated by Pax6, is expressed during DG formation and plays an essential role in neurogenesis [74]. Ngn2 constitutive knockout mice exhibit reduced cell proliferation and failure to form the infrapyramidal blade of the DG. Interestingly, many promoters bound by Pax6 are also occupied by Sox2, suggesting that they function together in the regulation of NSCs maintenance and proliferation.

In addition to Sox2, Tlx, Pax6 and Ngn2, an additional transcription factor that is fundamental for preserving the NSC pool is the restrictive element-1 silencing transcription factor (REST), also known as neuron-restrictive silencing factor (NRSF), which acts as a transcriptional repressor by recruiting other corepressor, such as mSin3A/B [75], N-CoR, CtBP [76] or CoREST, on the promoter of coding and non-coding target genes [77]. REST plays a fundamental role both at level of neurogenesis and in postmitotic neurons where it fine-tunes the expression of genes important for synaptic plasticity. During neurogenesis, REST is important for keeping the neuronal genes repressed and therefore preserves the undifferentiated state of the NSC pool [78]. Moreover, REST is also involved in shaping the synaptogenesis in adult neurons by regulating the expression of several synaptic proteins, including GluN2B and KCC2.

Overall, the NSCs pool in the adult hippocampus is maintained in part by the integration of extrinsic signals including Notch and the canonical Wnt pathway, which both directly control the expression of *Sox2*. Sox2 on the other hand, exerts dual gene regulatory impacts, specifically by activating the expression of Tlx and Shh pathway and repressing the expression of the prodifferentiation genes, such as NeuroD1.

### 3.2. Transcriptional Control of Differentiation, Survival and Integration of Dentate Granule Neurons

As intermediate progenitors exit from the cell cycle, they commit to neuronal lineage and then differentiate into glutamatergic granule neurons; there is a switch in the transcriptional program to control the later stages of adult neurogenesis. One of the key transcription factors that regulates this transition is NeuroD1. NeuroD1 is expressed at high levels in several areas of the adult brain including hippocampus, OB and cerebellum. NeuroD1 expression is both under the control of canonical Wnt signalling and GABA neurotransmitter and its expression promotes neuronal differentiation of NSCs in vivo [79]. Generation of the first NeuroD1 constitutive knockout mouse shows a disorganization of the DG indicating the essential role of NeuroD1 in regulating hippocampal development [80]. This first evidence was also confirmed by the conditional deletion NeuroD1 in the adult DG [81]. In these mice, newborn granule neurons are markedly reduced due to failing in survive and integrate into the pre-existing network.

In immature granule neurons, NeuroD1 is co-expressed with the prospero related homeobox gene Prox1 [82] and its overexpression promotes neuronal differentiation of NSCs. Moreover, conditional ablation of Prox1 results in the alteration of DG structure. In particular, the infrapyramidal blade fails to form and the size of the suprapyramidal blade is reduced [83]. Although most of the transcription factors are transiently expressed during neurogenesis, Prox1 expression seems to persist also in mature granule neurons. Indeed, conditional deletion of Prox1 in newly generated mature neurons affects Calbindin expression [84]. Overall, NeuroD1 and Prox1 appear to be a key player in regulating the differentiation of dentate granule neurons.

Sox family may also participate in the regulation of the transition from NPCs to immature neurons. Sox3 is closely related to Sox1 and Sox2 gene and is expressed in the early CNS. Consistent with this pattern of expression, constitutive deletion of Sox3 in mice results in congenital abnormalities in the hippocampus and corpus callosum [85]. Sox11 is mainly expressed in neurogenic niches of the adult mammalian brain and its ectopic expression promotes neuronal differentiation of NPCs [86]. In addition, in vivo conditional ablation of Sox11, by stereotactic injection of the Moloney Murine Leukemia Virus (M-MLV) CAG–GFP–IRES–Cre into the dentate gyrus of adult Sox4/Sox11dcKO mice inhibits neurogenesis without affecting proliferation and survival of NPCs. Mechanistically, Sox11 directly regulates the expression of the neuronal lineage-specific gene DCX [87]. In addition, Sox11 also modulates dendrites development in adult DG neurons [88].

Moreover, FoxG1 is a Winged-Helix transcriptional repressor mainly expressed in telencephalon and in the adult DG, and plays an important role in postnatal hippocampus neurogenesis. In fact, FoxG1 haploinsufficiency results in the reduction of hippocampal volume and granule cell number with memory deficits [89]. Moreover, conditional deletion of Foxg1 leads to developmental defects in the adult DG, characterized by the loss of the SGZ [90]. These abnormalities are caused by a defect in self-renewal, differentiation and migration of newly generated neurons.

The last stage of hippocampal neurogenesis, maturation ad integration of granule neurons is mainly under the control of the transcription factor cAMP response-element binding protein (CREB1). CREB1 is a member of the bZIP superfamily of transcription factors that plays an important role in modulating neuronal survival, maturation and plasticity in the adult DG [91]. In particular, GABA-mediated excitation increases CREB signalling and results in the enhancement of dendrite length and an increase in dendritic branching [92]. Conversely, when CREB function is lost by in vivo retrovirus-mediated genetic manipulation into adult brain, maturation and complexity of dendritic tree is impaired and the expression of the neurogenic transcription factor NeuroD1 is decreased [93]. In addition, it has been shown that CREB regulates the survival of newborn neurons. However, it should be noted that CREB mutant mice have increased levels of hippocampal neurogenesis [94,95].

### 3.3. The Well-Established Role of p53-Family During Hippocampal Neurogenesis

The family of transcriptional factors p53, p63 and p73 [96,97,98,99,100,101,102] are well known and characterised for their role in the control of the cell cycle arrest and apoptosis [103,104,105,106,107,108,109,110,111]. Together with the frequent p53 mutations in cancer [112,113,114,115,116,117,118,119,120,121,122], this signalling leads to associate their function to oncosuppression [123,124,125,126]. There is, however, strong in vivo and in vitro evidence that the family is also implicated in the regulation of the CNS functions [127,128,129,130,131]. Mechanistically, the p53-family proteins control NSCs survival, self-renewal and terminal differentiation via a complex transcriptional regulatory network by binding to specific DNA sequences to regulate the expression of coding- and non-coding genes.

p53 mRNA expression is mainly confined to an in area of the developing brain that does not undergo apoptosis, suggesting that p53 participates in neuronal differentiation [132,133,134]. The role of p53 in neuronal differentiation is further supported by in vivo studies. p53-null mice, in particular female, display exencephaly due to an overgrowth of neural tissue, which in turn, causes failure of the neural tube to close during brain development [135]. p53 controls proliferation and differentiation of NPCs both in vitro and in vivo mainly by regulating the expression of several cell-cycle regulators without affecting the expression of the canonical neuronal markers [136,137]. In post-mitotic neurons beyond the regulation of neuronal apoptosis, post-translational modifications of p53 (acetylation) might promote neuronal maturation and axonal regeneration [138]. p53 has also been associated to phosphorylation of tau protein in the pathogenesis of the Alzheimer’s disease [139].

Among the members of the p53 family, p73 is a key player in the regulation of CNS development and function by modulating NSC self-renewal and differentiation as well as promoting terminal neuronal differentiation [140,141,142]. The Trp73 gene, codifying the p73 proteins, has a complex structure, including two alternative promoters that regulate expression of the N-terminal full-term isoform, TAp73, and the N-terminal truncated ΔNp73 isoform. The crucial role of p73 in the CNS has been highlighted by the phenotype of the several knockout mice for p73: global p73KO mice and the isoform-specific TAp73 or ΔNp73 models [101,143] [144,145,146,147]. Indeed, deletion of both isoforms results in hippocampal dysgenesis that is characterized by the partial or total loss of the lower blade of the DG and by an impaired organization of CA1 and CA3 regions [140]. Moreover, p73 is essential for maintaining the neurogenic pool in SVZ and SGZ by promoting self-renewal and proliferation and inhibiting premature senescence of NSCs and/or NPCs [148,149,150]. Mechanistically, TAp73 either directly or indirectly regulates the expression of genes involved in metabolism [151,152,153] and NSCs maintenance including, Sox2, Sox3, TRIM32 and Notch signalling pathway. Similarly, TAp73 regulates NSCs maintenance in the olfactory bulb by transcriptionally regulating the expression of Hey-2 [154]. TAp73 is also implicated in the regulation of post-mitotic neurons, by modulating expression of the neurotrophin receptor p75 (p75^NTR^), which is implicated in axonal growth and dendritic arborisation [155,156]. Moreover, TAp73 controls neuronal terminal differentiation by regulating the expression of synaptic proteins such as synaptotagmin-1 and syntaxin-1A via miR-34a [157,158,159].

**Figure 3:**
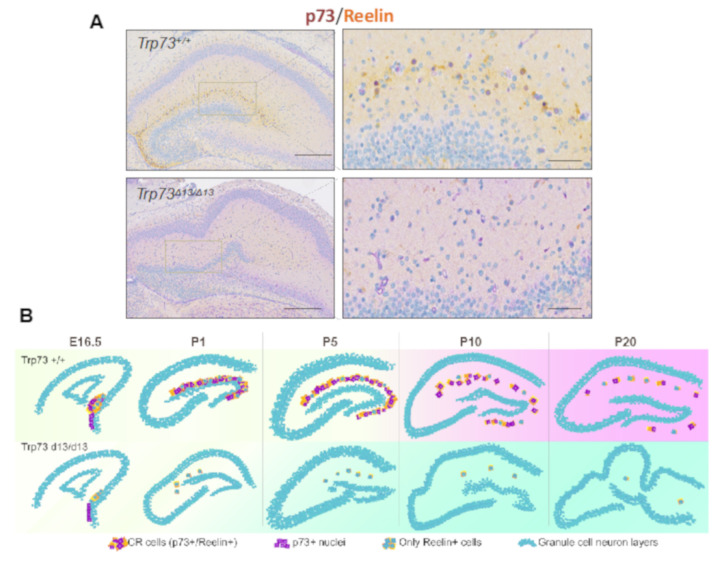
Trp73^D13/D13^ mice display hippocampal dysgenesis and CR cells depletion. (**A**) Immunohistochemistry of postnatal day 5 (P5) mouse hippocampus displays disrupted morphology and reduced presence of Reelin+ CR cells in Trp73^D13/D13^ genotype. Scale bars indicate 500 µm and 50 m.(**B**) Representative summary of morphological developmental progression in the hippocampus of Trp73+/+ and Trp73^D13/D13^ from embryonic 16.5 (E16.5) to postnatal day 20 (P20) stage. Adapted with modifications from Amelio et al. [162].

The recently described Trp73^d13/d13^ mice, lacking exon 13 in the p73 gene, have elucidated p73 C-terminus contribution to brain development. p73 C-terminus can undergo a complex alternative splicing, which can give rise up to 7 different isoforms (α, β, γ, σ, ε, ζ, η) [160,161]. Deletion of exon 13 produces a switch of the longest and most expressed isoform α into the isoform β, which in contrast to α does not contain the sterile alpha-motif (SAM) domain. Replacement of α with β substantially affects brain development, producing hippocampal dysgenesis, which largely recapitulates the phenotype of the global p73KO and the selective TAp73 KO. In particular, Trp73^d13/d13^ mouse developing brain displays a progressive depauperation of Cajal–Retzius (CR) cells [162] (Figure 3). Thus, the hippocampal dysgenesis appears to be a consequence of deprivation of the CR cells, which are physiologically deputed to direct brain architecture during embryonic development.

## 4. Metabolic Regulation of Adult Neurogenesis

The mammalian neurons strictly depend on glucose as the main source of energy and energy metabolism is tightly regulated during neuronal differentiation [163,164,165,166] and degeneration [167]. Neurons rely on oxidative phosphorylation (OXPHOS) to meet energy demands, e OXPHOS, neurons metabolise one glucose molecule to obtain 30–32 ATP molecules of energy. Therefore, mitochondria play a key role during neurodevelopment and adult neurogenesis for cytoskeletal remodelling, outgrowth of axons, dendrites and synaptic activity [168,169]. Peroxisome proliferator-activated receptor gamma coactivator 1 (PGC-1α) is a master regulator of mitochondrial biogenesis by activating the expression of nuclear respiratory factor 1 (NRF1), which controls the expression of mitochondrial proteins including cytochrome-c (cyt-c), and mitochondrial transcription factor A (TFAM). PGC-1α regulates the formation and maintenance of the synapsis in developing and adult hippocampal neurons [170].

Nuclear-encoded mitochondrial proteins are regulated by several factors such as oncogenes and tumour suppressor proteins [171], including p73, oestrogen-related receptor (ERRα), nuclear respiratory factors NRF1 and NRF2 and the yin yang 1 transcription factor (YY1). Indeed, TAp73 directly regulates the expression of the cytochrome C oxidase subunit 4 (COX4I1), a mitochondrial protein from the complex IV, which is essential for energy supply in neurons [172,173]. NRF2 deletion affects the proliferation capacity of NSCs from SGZ and impairs neuronal differentiation [174] and the psychiatric susceptibility gene Cacna1c also promotes mitochondrial resilience to oxidative stress in neurons [175].

Post-translation modification also regulates mitochondria function during neuronal differentiation. Cytoplasmic polyadenylation element-binding protein 1 (CPEB1) controls translation of NDUFV2 and complex I activity. Indeed, CPEB1 deficient neurons show reduced ATP production and impaired dendrite branching. This phenotype can be rescued by overexpressing NADH:Ubiquinone Oxidoreductase Core Subunit V2 (NDUFV2) [176].

Neuronal differentiation implies a substantial metabolic reprograming [177,178]: the switch from aerobic glycolysis to OXPHOS characterizes neuronal differentiation of NPCs [179]. In particular, the transition from NPCs to neurons is characterized by a reduced expression of hexokinase (HK2) and lactate dehydrogenase (LDHA), together with a switch in pyruvate kinase splicing from PKM2 to PKM1. In addition, neuronal differentiation is associated with the upregulation of the master regulators of mitochondrial biogenesis, PGC-1α, ERRγ and TFAM. Since mitochondrial mass increases proportionally with neuronal mass growth, the upregulation of mitochondrial biogenesis could in part support cell growth, i.e., axonal growth and dendritic development [180]. During terminal differentiation of cortical neurons, an increase in glucose metabolism is associated with a rise in glucose uptake and enhanced GLUT3 expression. Aerobic glycolysis in neurons sustains axonal elongation and synaptogenesis [181], regardless of the efficiency of the related energy production. This metabolic reprogramming is secondary to activation of the PI3K/mTOR axis [180]. Indeed, pharmacological inhibition by Rapamycin of the PI3K/mTOR axis results in a reduction of mitochondrial biogenesis and glucose metabolism in neurons that are associated with a reduction in terminal neuronal differentiation. Therefore, mTOR signalling plays a role in the regulation of mitochondrial mass and functions together with the regulation of glycolysis, which in turn is required for dendrite and synapse formation.

Glutamine is also an amino acid linked to cellular energy homeostasis. Glutamine can be converted to glutamate and then into α-ketoglutarate and thus be oxidized in the tricarboxylic acid (TCA) cycle to synthetize ATP. Terminal neuronal differentiation is also associated with increased glutamine metabolism, resulting in increased expression of neurotransmitters such as glutamate and GABA, and increased TCA cycle activity [180].

**Figure 4:**
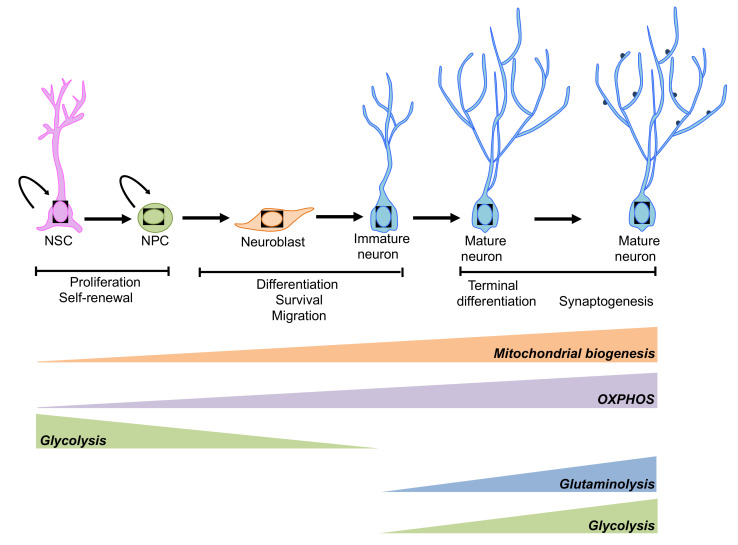
Schematic representation of the metabolic pathways involved in the regulation of neurogenesis. An extensive reprogramming of cell metabolism is associated with neurogenesis. NSCs and neuronal progenitor cells (NPCs) rely mainly on glycolysis, while mature neurons preferentially use OXPHOS. However, under some circumstances aerobic glycolysis and glutaminolysis has been observed during neuronal terminal differentiation (please see main text for details).

Overall, Figure 4, aerobic glycolysis and OXPHOS are the two main pathways providing metabolic precursors for biosynthesis and energy production. The activities of these pathways are tightly regulated to guarantee optimal resource supply, conforming to cellular function. The balance between aerobic glycolysis and OXPHOS is vital for neuronal development [182].

All together, these observations support a crosstalk between signalling and metabolism.

In the past 20 years, many significant questions have been addressed and some basic principles have emerged in the field of adult neurogenesis. However, more recently, the persistency of adult neurogenesis in adult human brain has been questioned [183]. The main challenge in studying human adult neurogenesis is the access to samples. Since the preservation of human brain will affect the labelling of new adult-born neurons, the creation of brain banks with standardised tissues collection and preservation is a main priority for studying adult neurogenesis. In addition, we need to have unanimous criteria for how to identify NSCs from progenitor cells. Moreover, single-cell RNA sequencing will also give fundamental information for understanding the cellular components in neurogenic niches.

## 5. Conclusions

A large body of evidence indicates that the alteration of neurogenesis is associated with several human pathologies and with age-associated decline in cognitive function [184]. Therefore, understanding the epigenetic, transcriptional and metabolic mechanisms underlying neuronal differentiation, and how these processes are deregulated, could open novel therapeutic strategies for treating brain disorders. Several HDAC inhibitors are neuroprotective in many mouse models of neurodegeneration [185] and several clinical trials are either ongoing or completed (NCT03056495 and NCT02124083). Moreover, modulation of metabolic pathways with small molecules might be an alternative possible novel therapeutic strategy in the near future. Indeed, mTOR signalling by regulating cell growth and metabolism is emerging as a crucial player in proliferation, differentiation, and neurite outgrowth and synaptic formation and its activity can be modulated by rapamycin/rapalogs [186,187]. Failures in mitochondria OXPHOS lead to several neurological disorders including Leigh syndrome, a severe childhood neurological disorder [188]. Rapamycin treatment of a mouse model of the syndrome delays onset of neurological symptoms reduces neuroinflammation and prevents brain lesions [189].

## Abbreviations

CaMKII	Calcium/Calmodulin-Dependent Protein Kinase II
CHD	Chromodomain Helicase DNA-Binding
CNS	Central Nervous System
COX4I1	Cytochrome C Oxidase Subunit 4
CPEB1	Cytoplasmic Polyadenylation Element-Binding Protein 1
Cspg4	Chondroitin Sulphate Proteoglycan 4
cyt-c	Cytochrome-C
DG	Dentate Gyrus
Dlx2	Distal-Less Homeobox 2
DNMTs	Dna Methyl Transferases
ERRα	Oestrogen-Related Receptor Alpha
Ezh2	Zeste Homologue 2
GADD45β	DNA-Damage-Inducible Protein 45β
Gal	Galanin
HATs	Histone Acetyltransferases
HDACs	Histone Deacetylases
hGFAP	Human Glial Fibrillary Acidic Protein
HK2	hexokinase
JMJD3	Jumonji Domain-Containing Protein 3
KMTs	Lysine Methyltransferases
LDHA	lactate dehydrogenase
LSD1	Lysine-Specific Demethylase 1
MLL1	Mixed-Lineage Leukaemia 1
M-MLV	Moloney Murine Leukemia Virus
Ngb	Neuroglobin
NPCs	Neuronal Progenitor Cells
NRSF	Neuron-Restrictive Silencing Factor
NSCs	Neural Stem Cells
OB	Olfactory Bulb
OXPHOS	oxidative phosphorylation
PRC	Pcg Repressive Complex
REST	Restrictive Element-1 Silencing Transcription Factor
SAM	sterile alpha-motif
SGZ	Subgranular Zone
Shh	Sonic hedgehog
SVZ	Subventricular Zone
TET	Ten-Eleven Translocation
TRXG	Trithorax Active Complex
YY1	Yin Yang 1 Transcription Factor
